# First multicenter study on multidrug resistant bacteria carriage in Chinese ICUs

**DOI:** 10.1186/s12879-015-1105-7

**Published:** 2015-08-21

**Authors:** Xiaojun Ma, Yinghong Wu, Liuyi Li, Qian Xu, Bijie Hu, Yuxing Ni, Anhua Wu, Shumei Sun, Vincent Jarlier, Jérôme Robert

**Affiliations:** Peking Union Medical College Hospital, Beijing, China; Peking University People’s Hospital, Beijing, China; Peking University First Hospital, Beijing, China; China-Japan Friendship Hospital, Beijing, China; Zhongshan Hospital Fudan University, Shanghai, China; Ruijin Hospital, Shanghai Jiaotong University School of Medicine, Shanghai, China; Xiangya Hospital, Central South University, Changsha, China; Nanfang Hospital, Southern Medical University, Guandong, China; Pitié-Salpêtrière University Hospital, Assistance Publique des Hôpitaux de Paris (APHP), Paris, France; Sorbonne Universités, UPMC Univ Paris 06, CR7, INSERM U1135, Centre d’Immunologie et des Maladies Infectieuses, CIMI, Team E13 (Bacteriology), F-75013 Paris, France

## Abstract

**Background:**

The importance of multidrug-resistant organisms (MDRO) in Chinese hospitals is not clearly delineated. Thus we sought to assess the prevalence of MDRO in Chinese intensive care units (ICUs).

**Methods:**

Prospective study of inpatients admitted consecutively to eight ICUs in four Chinese cities in 2009–10. Admission and weekly screenings were performed by using selective media for methicillin resistant *Staphylococcus aureus*, extended-spectrum beta-lactamase-producing Enterobacteriaceae, *Acinetobacter* and *Pseudomonas aeruginosa*. For the two latters, resistance to ceftazidime defined MDRO. Backward logistic regression models were designed to assess factors independently associated with MDRO carriage on admission and MDRO acquisition within ICUs.

**Results:**

686 patients were included, and the MDRO prevalence rate on admission was 30.5 % (32.7 % for ESBL-positive Enterobacteriaceae, 3.2 % for MRSA). Antibiotic treatment prior to ICU admission was independently associated with carriage on admission (OR: 1.4) in multivariate analysis. A total of 104 patients acquired ≥1 MDRO in ICU (overall attack rate: 23.7 %; 14.9 % for ESBL-positive Enterobacteriaceae, and 5.1 % for MRSA). The MDRO attack rate increased from 13.2 % in the first week to 82.1 % for ICU stay > 3 weeks. Duration of antibiotic exposure (OR: 1.16; 1.1–1.2) and prior antibiotic treatment before ICU (OR: 2.1; 1.1–3.3) were associated with MDRO acquisition in multivariate analysis. The MDRO prevalence rate on ICU discharge was 51.2 % and the global prevalence density rate 71 per 1000 hospital-days.

**Conclusion:**

More than one out of two patients was MDRO carrier on ICU discharge in Chinese hospitals. This is the result of the combination of a high MDRO prevalence rate on ICU admission and a high MDRO acquisition rate within ICU.

## Background

Multidrug-resistant organisms (MDRO) are a threat throughout the world due to antibiotic overuse and cross-transmission [1]. Infections due to MDRO increase morbidity and even mortality among inpatients. Intensive care units (ICU) are the wards where antibiotic resistance and antibiotic use are the highest, and where the impact of antibiotic resistance is the highest [2, 3]. Accordingly, actions to limit the rise of MDRO within hospitals should focus on ICUs. Such programs aimed at decreasing the burden of multidrug resistance were developed in many countries, and have been shown to prevent cross-transmission within hospitals [4–6]. In China, infection control teams are in place in hospitals since 2000, but isolation precautions are seldom implemented. However, it is only very recently that policies regarding antibiotic use in hospitals have been issued.

Data on the epidemiology of MDRO in healthcare in Mainland China are scarce. Most of the reports are dealing on identification of resistant mechanisms in Gram-negative species such as extended-spectrum beta-lactamases (ESBL), metallo-betalactamases, or OXA-type beta-lactamases [7–11]. Few recent data report on the epidemiology of MDRO in the country. Recently, comparisons of the prevalence of MDR *Acinetobacter baumannii* between Hong-Kong and other regions of China showed that Hong-Kong had the lowest prevalence [12]. A national antimicrobial resistance investigation network (CHINET) [13, 14] has conducted multicentre studies in 15 cities under the coordination of the Chinese Ministry of Health (MOH). Methicillin-resistant *Staphylococcus aureus* (MRSA) have been shown to represent 62.9 % of all *S. aureus* isolates isolated during the study period, and 47.5 % of *Escherichia coli* isolates and 29.2 % of *Klebsiella pneumoniae* isolates were resistant to cefotaxime [13]. However, there are some methodological issues. Indeed, one may question in China if patients with the most difficult to treat diseases or with relapsing infections are not more likely to be sampled than patients improving with a short course of empiric antibiotic regimen. Consequently, available data on bacterial resistance may overestimate resistance rates.

Because of the lack of data at the country level, and because it is of paramount importance to assess the magnitude of antimicrobial resistance to evaluate the current situation, and help promoting a program to curb MDRO in China, we designed a multicentre study to evaluate the baseline prevalence rates of MDRO in a sample of Chinese ICUs.

## Methods

### Surveillance program

A surveillance and prevention program of MDRO was designed in 2008 by the Chinese Ministry of Health with the technical support from Assistance-Publique Hôpitaux de Paris, France and the financial support of bioMérieux, France. The evaluation of the burden of MDRO in ICUs was chosen to initiate the program over a 6-month period in 2009–2010.

All patients consecutively admitted during the study-period to participating ICUs for a length of stay > 24 hours were screened on admission, weekly thereafter and on ICU discharge, for nasal carriage of MRSA and digestive carriage of ESBL-producing Enterobacteriaceae as well as *A. baumannii* or *Pseudomonas aeruginosa* resistant to ceftazidime. The duration of participation varied according to each ICU.

### Bacteriology

Nasal swabs were inoculated onto ChromID MRSA and rectal swabs onto ChromID ESBL (bioMérieux, Marcy l’Etoile, France). All green colonies growing on ChromID MRSA after 24 hours were considered as MRSA [15]. For each type of gram-negative bacilli isolated on the ChromID ESBL screening media, identification and antibiotic susceptibility testing were performed by Vitek2 (bioMérieux, France), and isolates suspected to produce ESBL were sent in a referral centre for ESBL confirmation by using ESBL Etest strips (bioMérieux, France). *A. baumannii* and *P. aeruginosa* isolates resistant to ceftazidime were considered as MDRO.

### Data collection

Data were prospectively collected and included basic demographic data on admission, previous history of hospitalization in the last 6 months, antibiotic and invasive devices use three month before and during admission. Patients harbouring MDRO in a clinical sample were also considered as MDRO carriers. The time to MDRO acquisition was calculated from ICU admission to the first MDRO-positive sample.

### Analysis

Data were analysed by using Stata 11 (StataCorp, College Station, TX). Categorical variables were compared using the Fischer exact test, and the Mann–Whitney test was used for continuous variables. Multivariate analysis was performed by using logistic regression to determine factors independently associated with ICU acquisition of MDRO among non-carriers and factors associated with carriage on admission. Variables with *p* < 0.10 in univariate analysis were introduced in the model, and backward analysis was performed. Variables not significantly associated with the outcome were removed based on the Wald statistic. The Hosmer-Lemeshov test was used for assessing models’ fitness. Only the most parsimonious models, i.e. those with the least variables and the most significance, are presented. Partially correlated variables were not introduced simultaneously in the models. P values are two tailed, and *P* <0.05 was considered statistically significant.

The prevalence rate was defined as the proportion of patients carrying a MDRO during the 6-month study period per 100 ICU admissions. The attack rate is a cumulative incidence rate and was defined as the proportion of patients acquiring MDRO within ICU during the study period per 100 patients admitted within ICU and included in the study. The incidence density rate was defined as the proportion of patients carrying a MDRO during the study period per 1000 hospital-days computed for patients included in the study.

Data collected were part of standard care during the period of the study, and were anonymysed before processing. Ethical clearance was obtained locally for each of the eight participating hospitals (Beijing:Peking Union Medical College Hospital, Peking University People’s Hospital, Peking University First Hospital, and China-Japan Friendship Hospital; Shanghai: Zhongshan Hospital Fudan University, and Ruijin Hospital; Changsha: Xiangya Hospital,; Guandong: Nanfang Hospital). 

## Results

A total of 8 voluntary Chinese ICUs (5 mixed, 2 surgical, and 1 medical ICU) from four regions (4 in Beijing, 2 in Shanghai, 1 in Changsha, and 1 in Guandong) participated anytime in the prevalence study. The number of patients included in each ICU during the study period varied from 22 to 137 (median, 86) for a total of 686 patients. The overall characteristics of the 686 patients included in the study are given in Table 1. A total of 59.0 % were male, and 75.5 % had severe or critical status on admission. The proportion of patients with mechanical ventilation was 55.5 %. The later proportion varied from 31.4–94.9 %, three units having a proportion higher than 50 %.

**Table 1:** Characteristics of the patients according to the multi-drug resistant organisms (MDRO) carrier status

Characteristic	Total	Type of MDRO carriage			
	patients	On admission	ICU-acquired	Not carrier	*P* value^*^
	*n* (%)	*n* (%)	*n* (%)	*n* (%)	
Total	686 (100.0)	247 (100.0)	104 (100.0)	335 (100.0)	
Male (yes)	405 (59.0)	149 (60.3)	63 (60.6)	193 (57.6)	0.59
Severe/critical status on admission	525 (75.5)	192 (77.7)	87 (83.7)	246 (73.4)	0.04
Previous hospitalization	190 (27.7)	121 (49.0)	46 (44.2)	125 (37.3)	0.21
Origin of patients before ICU^a^					
- community	28 (4.1)	12 (4.9)	5 (4.8)	11 (3.3)	reference
- emergency room	115 (16.8)	40 (16.2)	30 (28.9)	45 (13.4)	1.0
- other wards	494 (72.0)	173 (70.0)	61 (58.7)	260 (77.6)	0.21
- other hospitals	30 (4.4)	13 (5.2)	6 (5.8)	11 (3.3)	0.58
- others/ no data	16 (2.3)	7 (2.8)	2 (1.9)	7 (2.1)	1.0
Invasive devices before ICU	313 (45.6)	132 (53.4)	48 (46.2)	133 (39.7)	0.24
Invasive devices in ICU	640 (93.3)	233 (94.3)	102 (98.1)	305 (91.0)	0.02
- mechanical ventilation	381 (55.5)	135 (54.7)	66 (63.5)	180 (53.7)	0.08
- indwelling urinary catheter	629 (91.7)	224 (90.7)	98 (94.2)	307 (91.6)	0.39
- intravascular catheter	514 (74.9)	200 (81.0)	88 (84.6)	226 (67.5)	0.001
Antibiotic in the last 3 months	279 (40.7)	118 (47.8)	58 (55.8)	103 (30.7)	<0.001
Antibiotic on ICU admission	586 (85.6)	204 (82.6)	89 (85.6)	293 (87.7)	0.57
- > 1 drug	232 (33.8)	82 (33.2)	39 (37.5)	111 (33.1)	0.41
Antibiotic in ICU					
- none	37 (5.4)	17 (6.9)	5 (4.8)	15 (4.5)	reference
- 1 drug	314 (45.8)	107 (43.3)	33 (31.7)	174 (51.9)	0.34
- 2 drugs	210 (30.6)	69 (27.9)	28 (26.9)	113 (33.7)	0.56
- > 2 drugs (3–7)	125 (18.2)	54 (21.9)	38 (36.6)	33 (9.9)	0.04
- penicillins	8 (1.2)	3 (1.2)	0	5 (1.5)	0.60
- penicillins + inhibitors	192 (28.0)	72 (29.2)	46 (44.2)	74 (22.1)	<0.001
- cephalosporins 1st & 2nd G	155 (22.6)	51 (20.7)	13 (12.5)	91 (27.2)	0.002
- cephalosporins 3rd G /aztreonam	223 (32.5)	79 (32.0)	40 (38.5)	104 (31.0)	0.16
- carbapenems	140 (20.4)	61 (24.7)	30 (29.9)	49 (14.6)	0.001
- aminoglycosides	13 (1.9)	4 (1.6)	4 (3.9)	5 (1.5)	0.23
- fluoroquinolones	111 (16.2)	45 (18.2)	15 (14.4)	51 (15.2)	0.84
- glycopeptides	116 (16.9)	45 (18.2)	33 (31.7)	38 (11.3)	<0.001
- others	194 (28.3)	69 (27.9)	32 (30.8)	93 (27.8)	0.55
Type of discharge at 28 ICU days^b^					
- discharged from hospital	83 (12.1)	35 (14.2)	16 (15.4)	32 (9.6)	reference
- remain in ICU	36 (5.2)	16 (6.5)	16 (15.4)	4 (1.2)	0.001
- other ward	516 (75.2)	173 (70.0)	58 (55.8)	285 (85.3)	0.01
- death	45 (6.6)	22 (8.9)	10 (9.6)	13 (3.9)	0.44
	Median (range)	Median (range)	Median (range)	Median (range)	
Age (year)	66 (9–101)	65 (9–101)	66 (21–95)	67 (15–94)	0.99
Length of stay (days)					
- in hospital before ICU admission	6 (0 – >60)	7 (0 – >60)	4 (0 – >60)	7 (0 – >60)	0.05
- in ICU	4 (4 – >28)	4 (1 – >28)	10 (2 – >28)	4 (1 – >28)	<0.001
- before MDR acquisition	7 (3 – >28)	0	7 (3 – >28)	-	
Total antibiotic-days in ICU		3 (0 –27)	5 (0–21)	3 (0–25)	<0.001

^*^Comparing ICU-acquired MDRO patients to non carriers; ^a^Data are missing for 3 patients; ^b^Data are missing for 6 patients

**Fig. 1:**
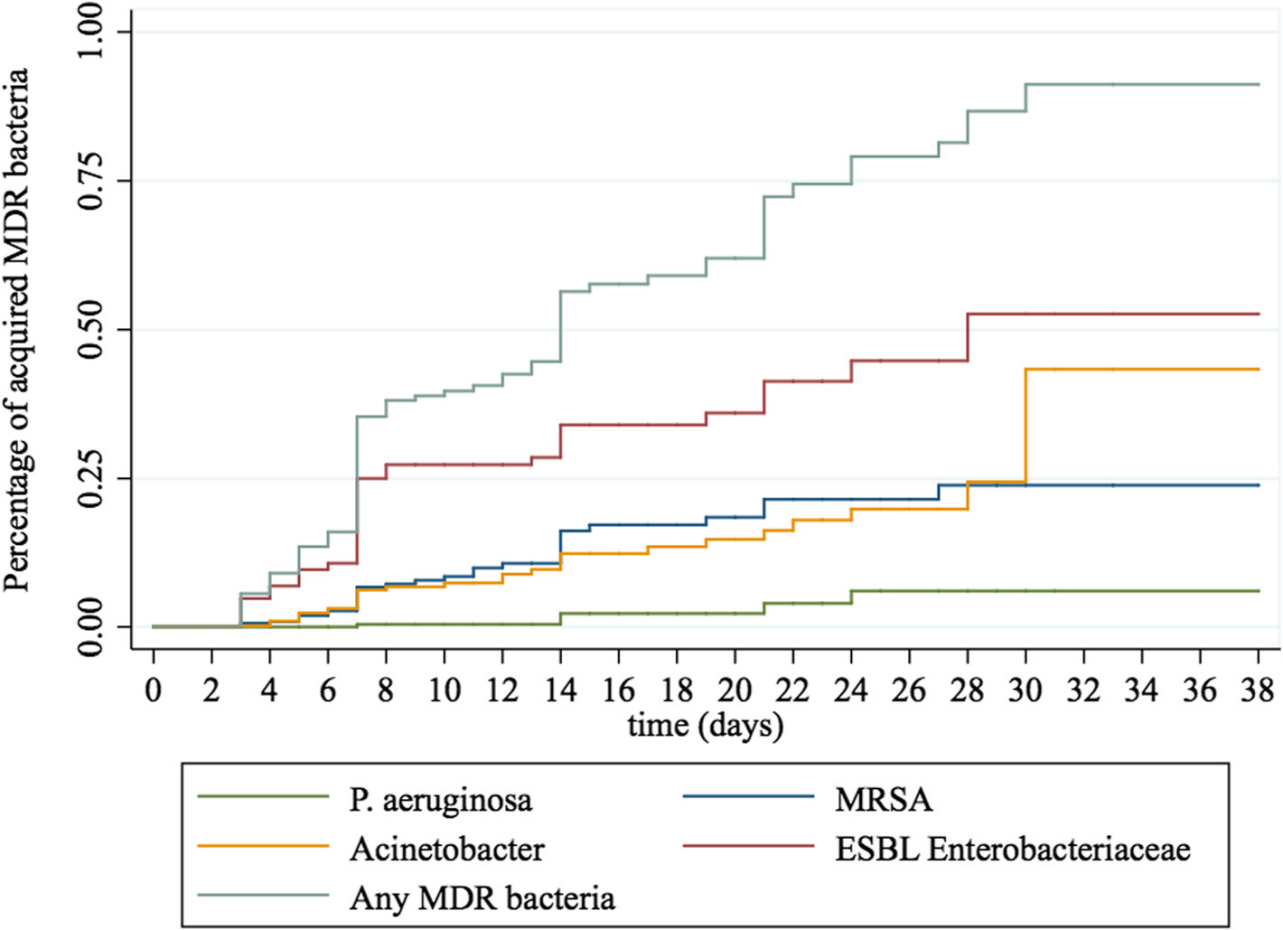
Kaplan-Meier curves representing multidrug resistant (MDR) organisms acquisition in intensive care units according to the length of stay

The global prevalence rate of MDRO on admission among all included patients (i.e. length of stay > 24 h) was 36.0 % (Table 2). The rates varied from 15.5–50.0 % according to the ICU, 6 out of the 8 ICUs units having rates > 25.0 % (Table 3). The rate was the highest for ESBL-producing Enterobacteriaceae (32.7 %), and the lowest for *P. aeruginosa* (0.6 %). Among all ESBL-producing Enterobacteriaceae isolated during the study period, 83.5 % belong to the *Escherichia coli* species, and 10.1 % were *Klebsiella pneumoniae*. A total of 104 patients acquired at least one of the four previously defined MDRO during ICU stay, resulting in an overall attack rate of 23.7 % and an incidence density rate of 35.0 per 1000 hospital-days (Table 2). The attack rates varied from 6.3–44.4 % and the incidence density rates from 12.3 to 45.8 per 1000 hospital-days according to the ICU (Table 3). The respective attack rates for each MDRO were 14.9 % for ESBL-positive Enterobacteriaceae, 5.4 % for MRSA, 5.2 % for *A. baumannii*, and 0.7 % for *P. aeruginosa* (Table 2). The overall prevalence rate on ICU discharge was 51.2 %, and the prevalence density rate of 71.1 per 1000 hospital-days. The median time of acquisition was 7 days for ESBL-producing Enterobacteriaceae, MRSA, and *A. baumannii*, and 14 days for *P. aeruginosa*. As expected, the risk to acquire MDRO increased with the ICU length of stay (Fig. 1). The overall attack rate increased from 13.2 % for patients hospitalized less than one week to 82.1 % for those staying more than 3 weeks (Table 2).

**Table 2:** Prevalence and incidence density rates of multidrug-resistant organisms (MDRO) in 8 Chinese intensive care units (ICU) for the 686 patients

Indicator	All MDRO	MRSA	ESBL-E	*A. baumannii*	*P. aeruginosa*
Number of imported cases	247	22	224	9	4
Number of ICU-acquired cases	104	36	69	35	5
Prevalence rate on admission (n/100 admissions)	36.0	3.2 %	32.7 %	1.3 %	0.6 %
Prevalence rate on discharge (n/100 admissions)	51.2 %	8.5 %	42.7 %	6.4 %	1.3 %
Global prevalence density rate (n/1000 hospital-days)	71.1 ‰	11.7 ‰	59.3 ‰	8.9 ‰	1.8 ‰
Incidence density rate (n/1000 hospital-days)	35.0 ‰	7.5 ‰	21.5 ‰	7.3 ‰	1.0 ‰
Attack rate (n/100 admissions)					
- overall	23.7 %	5.1 %	14.9 %	5.2 %	0.7 %
- ICU stay ≤ 1 week	13.2 %	2.1 %	9.4 %	1.8 %	0
- 1 week < ICU stay ≤ 2 weeks	40.3 %	11.5 %	26.2 %	12.2 %	0
- 2 weeks < ICU stay ≤ 3 weeks	56.5 %	19.5 %	21.4 %	15.9 %	0
- ICU stay > 3 weeks	82.1 %	13.7 %	46.7 %	11.8 %	9.6 %

*MRSA* methicillin-resistant *Staphylococcus aureus, ESBL-E* Enterobacteriaceae producing extended-spectrum beta-lactamase

**Table 3:** Prevalence and incidence density rates for all multidrug-resistant organisms (MDRO) according to the intensive care unit (ICU)

Rate	ICU identification							
	1 (*n* = 72)	2 (*n* = 78)	3 (*n* = 22)	4 (*n* = 132)	5 (*n* = 137)	6 (*n* = 90)	7 (*n* = 58)	8 (*n* = 97)
Number of beds	15	49	15	10	18	16	33	16
Annual number of admission	1400	3200	1200	1000	2400	2600	2200	1300
Median length of stay^a^ (range)	7.5 (2–33)	4 (2–28)	3.5 (2–17)	2 (2–28)	3 (2–38)	14 (2–29)	4 (2–22)	3 (2–28)
Number of imported cases	26	17	6	45	54	45	9	45
Number of ICU-acquired cases	19	9	1	12	19	20	7	17
Prevalence rate on admission (n/100 admissions)	36.1	21.8	27.3	34.1	39.4	50.0	15.5	46.4
Prevalence rate on discharge (n/100 admissions)	62.5	33.3	31.8	43.2	53.3	72.2	27.6	63.9
Attack rate (n/100 admissions)	41.3	14.8	6.3	13.8	22.9	44.4	14.3	32.7
Global prevalence density rate (n/1000 hospital-days)	64.5	58.2	55.1	95.8	114.1	47.5	47.2	85.6
Incidence density rate (n/1000 hospital-days)^a^	45.8	25.4	12.3	28.8	52.1	31.4	25.2	39.9
Median time acquisition (range)	7 (3–30)	4 (3–19)	7 (−)	7 (3–28)	5 (3–8)	7 (3–24)	7 (3–7)	12 (3–28)

^a^Length of stay for patients included in the study

Compared to patients with no MDRO on admission (Table 1), those carrying at least one MDRO on admission were more likely to have been hospitalized for a longer period of time before ICU admission (median 7 days versus 6 days, *p* = 0.05), to have a prior history of invasive device (53.4 % versus 41.2 %, *p* = 0.002), and to have received antibiotic in the last 3 months (47.8 % versus 36.7 %, *p* = 0.005). In the logistic regression analysis where all three latter variables were entered, prior history of antibiotic remained independently associated with MDRO carriage on admission (OR: 1.4; 95 % confidence interval: 1.1–2.0).

Compared to patients who did not acquire any MDRO during ICU (Table 1), those acquiring MDRO were more likely to have an intravenous catheter before MDRO acquisition (84.6 % versus 67.5 %, *p* = 0.001), to have received antibiotic in the last 3 months (55.8 % versus 30.7 %, *p* < 0.001), and to receive > 2 antibiotics in ICU (36.6 % versus 9.9 %, *p* = 0.04). Finally, patients with MDRO acquisition had a longer length of stay in ICU than those without MDRO (10 days versus 4 days, *p* < 0.001). In the final model of the logistic regression analysis, only duration of antibiotic exposure (OR: 1.16 for each additional day; 1.1–1.2) and prior antibiotic treatment before ICU admission (OR: 2.1; 1.3–3.3) remained significantly associated with MDRO acquisition. Other variables including severity status, presence of invasive devices, length of stay before ICU admission or within ICU did not remain significantly associated with MDRO acquisition.

In univariate analysis, factors associated with acquisition of either ESBL-producing Enterobacteriaceae (Table 4), or MRSA (Table 5), were comparable to those of any MDRO acquisition. Receiving a glycopeptide before MRSA acquisition was associated with a higher risk of MRSA acquisition (42.9 % versus 15.6 %, *p* < 0.001).

**Table 4:** Characteristics of the patients according to the carrier status of extended-spectrum beta-lactamase (ESBL) Enterobacteriaceae

Characteristics	ESBL-Enterobacteriaceae carriage			
	On admission	ICU-acquired	Not carrier	*P value* ^*^
	*n* (%)	*n* (%)	*n* (%)	
Total	224 (100.0)	69 (100.0)	393 (100.0)	
Male (yes)	137 (61.2)	45 (65.2)	223 (56.7)	0.19
Severe/critical status on admission	172 (76.8)	53 (76.8)	300 (76.3)	1.0
Previous hospitalization	111 (49.6)	36 (52.2)	145 (36.9)	0.02
Origin of patients before ICU^a^				
- community	10 (4.5)	4 (5.8)	14 (3.6)	reference
- emergency room	38 (17.0)	17 (24.6)	60 (15.3)	1.0
- other wards	155 (69.2)	42 (60.9)	297 (75.6)	0.27
- other hospitals	12 (5.4)	4 (5.8)	14 (3.6)	1.0
- others/no data	7 (3.1)	2 (2.9)	7 (1.8)	1.0
Invasive devices before ICU	117 (52.2)	29 (42.0)	167 (42.5)	0.94
Invasive devices in ICU	212 (94.6)	67 (97.1)	361 (91.9)	0.12
- mechanical ventilation	119 (53.1)	41 (59.4)	221 (56.2)	0.62
- indwelling urinary catheter	202 (90.2)	64 (92.8)	363 (92.4)	0.91
- intravascular catheter	182 (81.3)	56 (81.2)	276 (70.2)	0.06
Antibiotic in the last 3 months	104 (46.4)	37 (53.6)	138 (35.1)	0.003
Antibiotic on ICU admission	185 (82.6)	59 (85.5)	342 (87.2)	0.69
- > 1 drug	72 (32.1)	23 (33.3)	137 (34.9)	0.89
Antibiotic in ICU				
- none	16 (7.1)	3 (4.4)	16 (4.1)	reference
- 1 drug	98 (43.8)	27 (39.1)	189 (48.1)	0.72
- 2 drugs	64 (28.6)	19 (27.5)	125 (31.8)	0.72
- > 2 drugs (3–7)	46 (20.5)	20 (29.0)	63 (16.0)	0.55
- penicillins	3 (1.3)	2 (2.9)	5 (1.3)	0.31
- penicillins + inhibitors	62 (27.7)	27 (39.1)	104 (26.5)	0.03
- cephalosporins 1st & 2nd G	45 (20.1)	7 (10.1)	103 (26.2)	0.004
- cephalosporins 3rd G /aztreonam	75 (33.5)	28 (40.6)	122 (31.0)	0.12
- carbapenems	54 (24.1)	20 (29.0)	66 (16.8)	0.02
- aminoglycosides	4 (1.8)	1 (1.5)	8 (2.0)	1.0
- fluoroquinolones	41 (18.3)	8 (11.6)	66 (16.8)	0.28
- glycopeptides	39 (17.4)	17 (24.6)	60 (15.3)	0.05
- others	159 (71.0)	17 (24.6)	117 (29.8)	0.39
Type of discharge at 28 ICU days^b^				
- discharged from hospital	31 (13.8)	10 (14.5)	42 (10.7)	reference
- remain in ICU	16 (7.1)	10 (14.5)	10 (2.5)	0.02
- other ward	161 (71.9)	40 (58.0)	315 (80.2)	0.11
- death	15 (6.7)	5 (7.2)	25 (6.4)	1.0
	Median (range)	Median (range)	Median (range)	
Age (year)	65 (9 – 91)	64 (22–95)	67 (15–94)	0.60
Length of stay (days)				
- in hospital before ICU admission	7 (0 – >60)	5 (0 – >60)	6 (0 – >60)	0.77
- in ICU	4 (1 – >28)	8 (2 – >28)	4 (1 – >28)	<0.001
- before MDR acquisition	–	7 (3–28)	–	
Total antibiotic-days in ICU	3 (0–28)	4 (0–19)	3 (0–25)	<0.001

^*^Comparing ICU-acquired MDRO patients to non carriers; ^a^Data are missing for 3 patients; ^b^Data are missing for 6 patients

**Table 5:** Characteristics of the patients according to the carrier status of methicillin-resistant *Staphylococcus aureus* (MRSA)

Characteristics	MRSA carriage			
	On admission	ICU-acquired	Not carrier	P value*
	*n* (%)	*n* (%)	*n* (%)	
Total	23 (100.0)	35 (100.0)	628 (100.0)	
Male (yes)	12 (52.2)	21 (60.0)	372 (59.3)	0.93
Severe/critical status on admission	19 (82.6)	30 (85.7)	476 (75.8)	0.22
Previous hospitalization	11 (47.8)	11 (31.4)	168 (26.8)	0.54
Origin of patients before ICU^a^				
- community	2 (8.7)	1 (2.9)	25 (4.0)	reference
- emergency room	2 (8.7)	13 (37.1)	100 (16.0)	0.47
- other wards	17 (73.9)	18 (51.4)	459 (73.1)	1.00
- other hospitals	2 (8.7)	3 (8.6)	25 (4.0)	0.61
- others/no data	0	0	16 (2.6)	1.00
Invasive devices before ICU	16 (69.6)	14 (40.0)	283 (45.1)	0.56
Invasive devices in ICU	22 (95.7)	34 (97.1)	599 (95.4)	1.00
- mechanical ventilation	17 (73.9)	21 (60.0)	343 (54.6)	0.53
- indwelling urinary catheter	21 (91.3)	33 (94.3)	575 (91.6)	0.76
- intravascular catheter	17 (73.9)	32 (91.4)	465 (74.0)	0.03
Antibiotic in the last 3 months	16 (69.6)	23 (65.7)	240 (38.2)	0.001
Antibiotic on ICU admission	18 (78.3)	31 (88.6)	537 (85.7)	0.81
- > 1 drug	5 (21.7)	16 (45.7)	211 (33.6)	0.21
Antibiotic in ICU				
- none	2 (9.1)	0	32 (5.1)	reference
- 1 drug	10 (43.5)	10 (28.6)	294 (46.8)	0.61
- 2 drugs	4 (17.4)	7 (20.0)	195 (31.1)	0.60
- > 2 drugs (3–7)	7 (30.0)	18 (51.4)	107 (17.0)	0.03
- penicillins	0	0	10 (1.6)	1.00
- penicillins + inhibitors	8 (34.8)	15 (42.9)	172 (27.4)	0.05
- cephalosporins 1st & 2nd G	3 (13.4)	5 (14.3)	146 (23.3)	0.30
- cephalosporins 3rd G /aztreonam	5 (21.7)	15 (42.9)	205 (32.6)	0.21
- carbapenems	8 (34.8)	16 (45.7)	121 (19.3)	<0.001
- aminoglycosides	0	1 (2.9)	11 (1.8)	0.48
- fluoroquinolones	6 (26.1)	8 (22.9)	102 (16.2)	0.30
- glycopeptides	4 (17.4)	15 (42.9)	98 (15.6)	<0.001
- others	6 (26.1)	10 (28.6)	182 (29.0)	0.96
Type of discharge at 28 ICU days^b^				
- discharged from hospital	3 (13.6)	8 (22.9)	71 (11.3)	reference
- remain in ICU	2 (9.1)	6 (17.1)	28 (4.5)	0.27
- other ward	11 (50.0)	18 (51.4)	488 (77.7)	0.008
- death	6 (27.3)	3 (8.6)	36 (5.7)	1.00
Age (year)	71 (40–93)	70 (20–99)	65 (9–95)	0.11
Length of stay (days)				
- in hospital before ICU admission	14 (0 – >60)	3 (0 – >60)	6 (0 – >60)	0.12
- in ICU	7 (2 – >28)	12 (3–28)	4 (1 – >28)	<0.001
- before MDR acquisition	–	7 (3–27)	–	
Total antibiotic-days in ICU	6 (0–26)	6 (2–22)	3 (0–28)	<0.001

^*^Comparing ICU-acquired MDRO patients to non carriers; ^a^Data are missing for 3 patients; ^b^Data are missing for 6 patients

When considering MDR *A. baumannii* acquisition (data not shown), carriers were more likely than non-carriers to have had invasive devices before ICU admission (75.8 % versus 43.8 %, *p* < 0.001), and to have mechanical ventilation (84.9 % versus 53.6 %, *p* < 0.001). A separate analysis regarding MDR *P. aeruginosa* acquisition was not performed because of the low acquisition rate.

## Discussion

We conducted the first multicentre study on MDRO carriage in Chinese ICUs where systematic admission screening was not a general policy. We showed that almost one third of the patients carried MDRO on admission and that ESBL-producing Enterobacteriaceae were the most prevalent. There was a high incidence density rate (35 per 1000 hospital-days) resulting in one out of two patients carrying at least one MDRO on ICU discharge. MRSA incidence density rate was one third that of ESBL-producing Enterobacteriaceae. MDRO acquisition was significantly linked to the use of antibiotics.

The present study relied on systematic screening to assess the burden of MDRO in Chinese ICUs, as recommended in many countries [6, 16]. It has the advantage to identify asymptomatic carriers who are nevertheless disseminators. In our study, the overall MDRO carriage on ICU admission was rather elevated. The proportion of MRSA carriers on admission in our study (3.4 %) was slightly lower than in French ICUs at the end of the 1990s (4.2–6.9 %) [17, 18]. In studies conducted in western countries in the era of CTX-M ESBL-positive Enterobacteriaceae, carriage of such isolates was lower (2 % to 8 %) than in the present study [2, 19]. Nevertheless, the rate observed in Chinese ICUs is lower than the 60 % reported in an Indian ICU in 2008–2009 [20]. The high rate of MDRO carriers is likely to be a combination of poor hygiene and high antibiotic use before admission as demonstrated by the fact that, in our study, prior antibiotic exposure was independently associated with MDRO carriage. Cross-transmission may have occurred either in the community or within other wards before ICU admission [21]. However, no data are available to assess cross-transmission outside ICU in China, and data regarding the prevalence of MDRO in the community are also lacking.

The overall rate of MDRO acquisition was high. It ranged from 1 per 1000 patients-days for multiresistant *P. aeruginosa* to 21.5 per 1000 patients-days for ESBL-producing Enterobacteriaceae. The latter incidence rate is to be compared to those reported in France (1.6 to 5.3 per 1000 patients-days), or the Netherlands (14 per 1000 patients-days) [22, 23]. Incidence rates cannot be confronted to proportions of MDRO within each species, because the first relate mostly on carriage while the latters are only based on clinical samples. Incidence density data are drastically needed because it is a better estimate of the burden of MDRO as compared to proportions of resistant isolates within the species. However, the reported proportion of ESBL-producing isolates amongst *E. coli* and *K. pneumonia* isolated in clinical samples in China (56.2 % and 43.6 %, respectively) [13] is far higher than the same proportions reported in France (8 % and 13 %, respectively) or in the Netherlands (4 % and 6 %, respectively) [22, 23]. Therefore, findings of higher incidence density rates in China as compared to other countries are concordant with MDRO proportions reported elsewhere. Of interest, the median time of acquisition reported for ESBL-producing Enterobacteriaceae (7 days) was similar to the one reported in France where the incidence density rate is much lower [3]. We found that antibiotic exposure was an independent risk factor for MDRO acquisition. No antibiotic policy was implemented at the time of the study in participating ICUs as well as in most Chinese facilities. Moreover, data on antibiotic use in Chinese ICUs are missing preventing any benchmarking. Poor hygiene is likely to play a major role in MDRO cross-transmission within ICUs in addition to antibiotic exposure. However, because the present study was not designed to assess factors involved in cross-transmission, we did not collect factors known to be linked to acquisition such as hand hygiene or isolation precautions compliance, colonisation pressure, nurse-to-patient ratio, case-mix, and intensity of care. Of note, isolation precautions were not systematically implemented for MDRO carriers in participating ICUs.

Our study was not designed to assess carriage of *P. aeruginosa* or *A. baumannii* outside the digestive tract, although it has been demonstrated that both species are colonizing other sites [24]. It was considered that results from this first study would help to decide if more focused studies are needed. Therefore, rates reported herein are likely to represent the lower part of a range. However, the acquisition rate reported herein for *A. baumannii* is similar to that reported in Italy during an outbreak and should therefore be considered as worrisome [25]. Similarly, this study did not look for MRSA carriage outside anterior nares. Therefore MRSA rates reported in our study are likely to be slightly underestimated. Another weakness of our study is that we are not reporting the genetic mechanisms of resistance of MDRO. Although it is of interest to evaluate genes circulation around the country, it has not been demonstrated to be of any interest neither in the evaluation of the magnitude of the bacterial resistance issue nor in public health decision-making. Such data related to China may be found in other studies dedicated to this topic [7–10].

## Conclusion

The present study allowed evaluating the burden of MDRO in Chinese ICUs. We showed that antibiotic resistance is of concern in ICUs but also outside the ICU and mainly involved ESBL-positive Enterobacteriaceae. Our study is intended to be a baseline study to be repeated after implementation of control measures such as improving hygiene and mastering antibiotic use. Since our study, a new policy for antibiotic control was issued in China. This comprehensive policy includes restriction of antibiotic use, strengthening of laboratory diagnosis for bacterial resistance, antibiotic stewardship and building of regional surveillance networks. The effect of such a policy on MDRO rates could be evaluated by using a similar surveillance program.

## Abbreviations

ESBL: Extended-spectrum beta-lactamases
ICU: Intensive care units
MDRO: Multidrug-resistant organisms
MRSA: Methicillin-resistant *Staphylococcus aureus*
